# High-performance selective NO_2_ gas sensor based on In_2_O_3_–graphene–Cu nanocomposites

**DOI:** 10.1038/s41598-023-34697-5

**Published:** 2023-05-15

**Authors:** Alexander Khort, Yulyan Haiduk, Igor Taratyn, Dmitry Moskovskikh, Kirill Podbolotov, Alexandra Usenka, Natalia Lapchuk, Vladimir Pankov

**Affiliations:** 1 KTH Royal Institute of Technology, Teknikringen, 29, 114 28 Stockholm, Sweden; 2 Belarusian State University, Niezaleznasti av. 4, 220030 Minsk, Belarus; 3 Belarusian National Technical University, Prospekt Nezavisimosti, 65, 220013 Minsk, Belarus; 4 Center of Functional Nano-Ceramics, National University of Science and Technology MISIS, Lenin av. 4, 119049 Moscow, Russia; 5 Physical-Technical Institute, National Academy of Sciences of Belarus, Kuprevicha 10, 220141 Minsk, Belarus

**Keywords:** Nanoparticles, Sensors and biosensors, Nanoparticle synthesis

## Abstract

The control of atmosphere content and concentration of specific gases are important tasks in many industrial processes, agriculture, environmental and medical applications. Thus there is a high demand to develop new advanced materials with enhanced gas sensing characteristics including high gas selectivity. Herein we report the result of a study on the synthesis, characterization, and investigation of gas sensing properties of In_2_O_3_–graphene–Cu composite nanomaterials for sensing elements of single-electrode semiconductor gas sensors. The nanocomposite has a closely interconnected and highly defective structure, which is characterized by high sensitivity to various oxidizing and reducing gases and selectivity to NO_2_. The In_2_O_3_-based materials were obtained by sol–gel method, by adding 0–6 wt% of pre-synthesized graphene–Cu powder into In-containing gel before xerogel formation. The graphene–Cu flakes played the role of centers for In_2_O_3_ nucleation and then crystal growth terminators. This led to the formation of structural defects, influencing the surface energy state and concentration of free electrons. The concentration of defects increases with the increase of graphene–Cu content from 1 to 4 wt%, which also affects the gas-sensing properties of the nanocomposites. The sensors show a high sensing response to both oxidizing (NO_2_) and reducing (acetone, ethanol, methane) gases at an optimal working heating current of 91–161 mA (280–510 °C). The sensor with nanocomposite with 4 wt% of graphene–Cu additive showed the highest sensitivity to NO_2_ (46 ppm) in comparison with other tested gases with an absolute value of sensing response of (− ) 225 mV at a heating current of 131 mA (430 °C) and linear dependence of sensing response to NO_2_ concentration.

## Introduction

Detection and monitoring of concentrations of specific inorganic gases and volatile organic compounds (VOCs) in the local atmosphere are important tasks that are in high demand in many industrial processes, agriculture, environmental applications, and early diagnosis and monitoring of several diseases^1–5^. Furthermore, indoor air quality control is a necessary aspect of the improvement of living and working conditions. Thus, there is a constantly growing interest in the creation of advanced materials with enhanced gas-sensing properties. In this regard, nanoscale semiconductor metal oxides like SnO_2_, In_2_O_3_, WO_3_, and ZnO are considered among the best sensing materials for the detection of both oxidizing (Cl_2_, NO_2_, O_3_, CO_2_, etc.), reducing (CO, H_2_, SO_2_, CH_4_, etc.) and VOCs (acetone, ethanol, methane, etc.) gases^6–10^. Moreover, indium oxide, in particular, is a highly attractive material for the production of sensitive elements of semiconductor gas sensors due to its relatively low conduction activation energy. The combination of these factors enables the creation of sensing elements with a higher surface area and a number of reactive sites. For this reason, In_2_O_3_ in pure form and in combination with other oxides and additions of noble and transition metals has recently been intensively studied as material for gas sensors. As a result, a significant proportion of modern commercial semiconductor sensors are based on indium oxide^11–14^. However, because of a trend for device miniaturization and increasing demand for materials with higher performance, there is a need for improvement of characteristics of sensors, like lowering limits of detection and improving selectivity, which sets special requirements for the creation of new advanced materials and compositions.

The improvement of gas sensors based on In_2_O_3_, as well as sensors based on other oxides, is mainly associated with the development of nanostructured materials with different particle morphologies and their combinations with each other and conventional 3D nanocrystals. For instance, such structures as nanowires^15^, nanoribbons^16^, ultrathin nanotubes^17^, two-dimensional flakes^18^, and some others have already been used in sensors. However, only a limited number of semiconductor oxides could be or were obtained in such advanced morphology^19^. Another way to improve oxides’ sensing performance is their surface functionalization with materials and structures with a high specific area, and enhanced charge transfer properties like graphene and graphene-based materials.

It was shown, that graphene in its pure form shows gas-sensing properties even at room temperature^20^. However, the sensitivity of semiconductor sensors made of individual graphene or graphene oxide is low and has no commercial interest. The main reason for graphene’s poor sensing performance is in low defectiveness of its structure and the small number of free unsaturated bonds necessary for the physical and chemical adsorption of gas molecules. On the contrary, the presence of graphene and graphene-like materials in oxide compositions can significantly enhance their sensory properties^21–23^. The main reason for such a phenomenon considers the probable formation of electronic p–n-heterojunctions or p–p-homojunction with p- and n-type semiconductor oxides, respectively, which leads to the manifestation of a synergistic effect between the components of the composite and enhances the sensory response^24,25^. The nanocomposites based on graphene and its compositions with other materials like polymers and oxides have already been studied for gas sensing applications and showed promising results^26–29^.

The increased sensitivity of the composition of indium oxide with graphene oxide to a number of gases, especially nitrogen dioxide, was demonstrated before^30^. It has also been reported that metal nanoparticles integrated into a graphene matrix promote the formation of adsorbed oxygen ions and thus may have an additional effect in increasing the sensitivity of the composition^31–33^. A similar positive effect can also be expected from the presence of a small addition of transition metal oxides with p-type conductivity, such as, for example, CuO, in the composition with an n-semiconducting oxide^34,35^.

In this study, we report and discuss the result of an investigation of phase composition, microstructure, and gas sensing properties of In_2_O_3_ nanopowders, modified with defected and functionalized graphene–Cu (G@Cu) nanocomposites, obtained via solution combustion synthesis (SCS). The materials were prepared by adding 0–6 wt% of the G@Cu nanocomposite into the gel of the In-containing precursor. The synthesis approach led to the formation of closely interconnected In_2_O_3_–G@Cu structures with enhanced gas-sensing properties.

## Experimental

### Synthesis of materials

**Figure 1:**
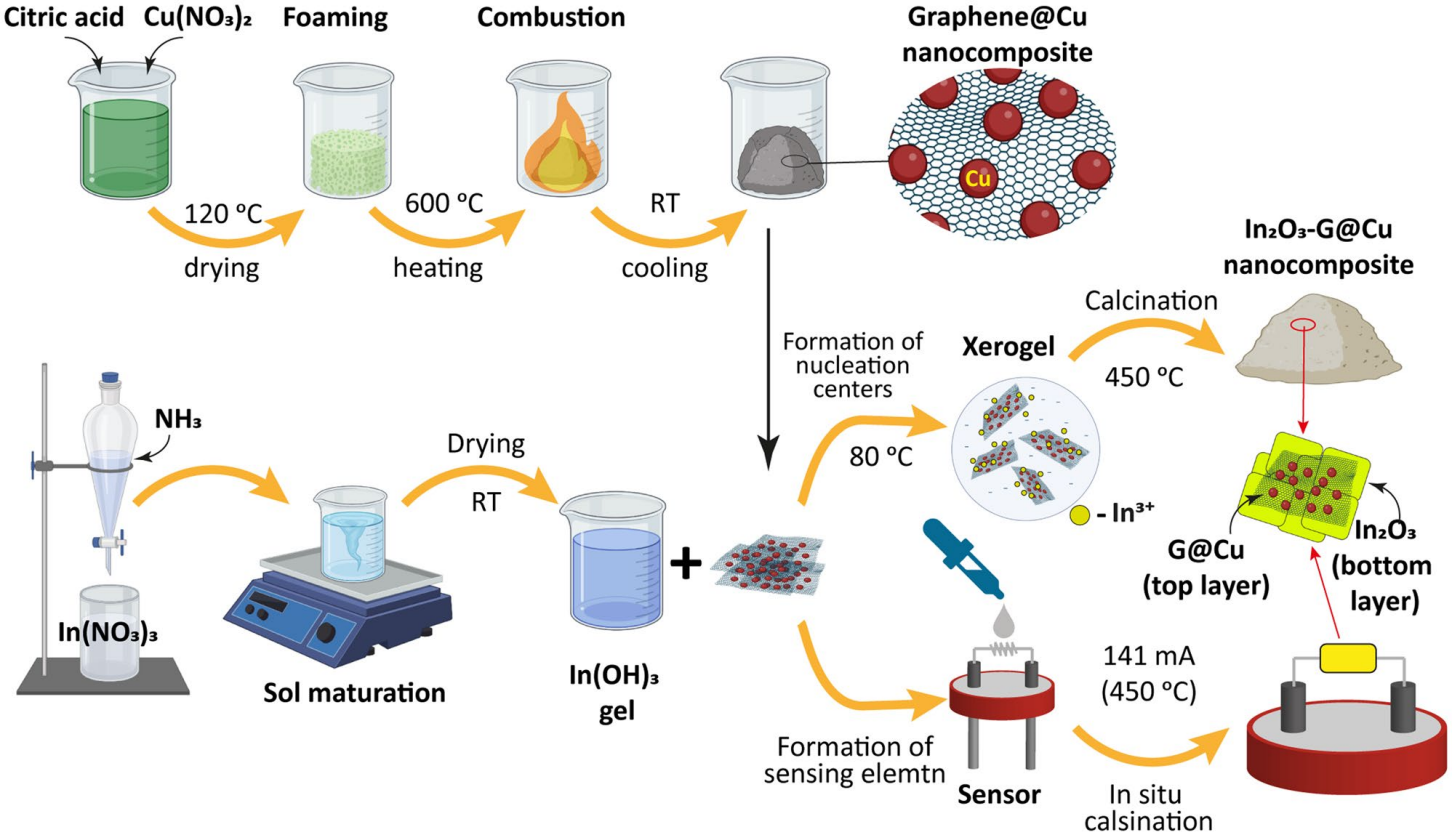
Simplified scheme of the In_2_O_3_–G@Cu powder nanocomposite synthesis and formation sensing element in situ.

The simplified scheme of the synthesis procedure of the experimental materials is shown in Fig. 1.

All raw materials used are of analytical grade quality and were used as there are without additional purification.

#### Preparation of In(OH)_3_ gel

The indium oxide, In_2_O_3_, was obtained by a sol–gel method according to the procedure described in our previous paper^25^. In brief, ~ 50 mL of 9.24 M aqueous solution of NH_3_ was dropwise added into 50 mL 0.38 M aqueous solution of indium nitrate (In(NO_3_)_3_·4.5H_2_O). The sol formation started at a pH of ~ 6–7 and finished at a pH of ~ 9. The sol was magnetically stirred for 30 min to equalize the concentration and for maturation. After that, the sol was washed several times using decantation to remove all electrolyte impurities and dried in air at room temperature to form an indium hydroxide (In(OH)_3_) gel.

#### Synthesis of the graphene–Cu composite

The graphene–Cu composite (G@Cu) was prepared by a modified SCS approach as described in^33,36^. For the preparation of 1 g of the composite 3.78 g of copper nitrate trihydrate (Cu(NO_3_)_2_·3H_2_O) as Cu source and oxidizer, and 12.23 g of citric acid monohydrate (C_6_H_8_O_7_·H_2_O) as carbon source and reducer, were dissolved in a minimal volume of hot distilled water. The solution was dried at 120 °C until xerogel had formed. The xerogel was calcinated in a preheated furnace at 600 °C in air, where a fast and highly exothermic combustion reaction occurred with the formation of the G@Cu nanopowder (Fig. S1) with a C:Cu mass ratio of ∼ 1:0.5–1. The detailed description and characterization of the obtained G@Cu composite is presented in our previous publications^33,36^.

#### *Preparation of In*_*2*_*O*_*3*_*–G@Cu composite*

To prepare the In_2_O_3_–G@Cu composite, the G@Cu powder in the amount required to obtain powders with the G@Cu concentration in a final material of 0 (pure In_2_O_3_), 1, 2, 3, 4, and 6 wt% (samples IG0, IG1, IG2, IG3, IG4, and IG6, respectively) was mixed with the In(OH_3_) gel. The gel-powder mixture was bath sonicated for 2 min and after that dried at 80 °C to form a xerogel. The final In_2_O_3_–G@Cu composite powder was obtained by calcinating the xerogel at 450 °C for 2 h. The powder then was manually milled in for further characterization.

#### Preparation of gas sensing elements

In this research, a single-electrode gas sensor with Pt spiral wire with a diameter of 20 μm was chosen for the study of gas sensing properties of the In_2_O_3_–G@Cu composites^19^. The schematic view and SEM image of the sensor, and measurement system diagram are presented in Figs. S2a,b, and S3, respectively. To prepare the sensing elements, the In(OH)_3_ gel with G@Cu powder was diluted with water, and a probe of the gel was placed on the Pt spiral wire, forming an oxide-based coating. The thickness of the metal oxide coating is in the range of 30–200 μm, and the length of the sensitive element is ~ 300 μm. After that, the gel was dried by applying the heating current of 120 mA (~ 370 °C) to the Pt electrode and then calcinated for 2 h in the air in situ by applying a heating current of 141 mA, which corresponds to the temperature of ~ 450 °C on the surface of the electrode. The calcination regime, in this case, is similar to the regime for obtaining powders.

### Characterization

#### Scanning electron microscopy (SEM) and energy-dispersive X-ray spectroscopy (EDS)

SEM and EDX were used to study materials microstructure and elements distribution using Mira 3 Tescan electron microscope equipped with Oxford Instruments X-Max 80 EDS system. SEM imaging and EDS mapping analyses were performed at an acceleration voltage of 15 kV.

#### X-ray powder diffraction (XRD)

XRD analysis was conducted using a Bruker D8 XRD Advance diffractometer (CuK**α**-radiation, α = 1.5405 Å). The XRD data were processed with High Score Plus software. Sherrer's equation was used for the calculation of the crystallite sizes. The analysis of the XRD patterns was made using the reference data from the COD2021 database.

#### Fourier-transform infrared (FTIR)

FTIR spectra were recorded on a Tenzor 27 Fourier spectrometer (Bruker) at room temperature using the KBr pellet method. The processing of the spectra was carried out using the OPUS 7.5 software package.

#### Transition electron microscopy (TEM)

The structure of the powders was studied using a JEOL JEM 2100 Plus transmission electron microscope (Japan). Sample preparation was carried out using a two-beam scanning electron microscope Helios G4CX FEI (USA). Powders were dispersed in isopropyl alcohol (C_3_H_8_O) using an ultrasonic bath for 15 min. Droplets of suspension were drop-cast onto support films grid (TedPella) and dried. The measurements were carried out using an accelerating voltage of 200 kV with spot size 1, alpha 2, and the condenser lens 1 (150 µm).

#### Electron paramagnetic resonance (EPR)

EPR spectra of the experimental samples were recorded at room temperature on a RadioPan SE/X–2543 with an H102 resonator in the X-band spectrometer operating at 100 kHz with an amplitude of 1 G and microwave radiation frequency of about 9.3 GHz. The EPR method enables the detection of paramagnetic defects in various structures containing unpaired electrons. The method is highly sensitive even to small amounts of impurities that affect the electronic structure of the main phase.

#### Spesific syrface area

The BET (Brunauer Emmet Teller) specific surface area of the nanocomposites was studied using Chemisorb 2750 (Micrometrics, USA). A load of ~ 0.1 g of powders were placed in a quartz test tube. To remove adsorbed impurities, annealing was carried out in a helium flow (50 mL/min) at 300 °C for 20 min. During measurements mixture of nitrogen (30 vol%) and helium of 12 mL/min was purged through the test tube. The adsorption capacity was determined in the sorption and desorption modes: the sample was cooled to 77 K (the boiling point of liquid nitrogen), recording the absorption of nitrogen from the gas mixture, and after reaching a steady state, the test tube the sample was warmed to room temperature and the amount of nitrogen released by the material was recorded.

### Gas sensing properties

The sensing response of the sensors under different conditions was studied using a home-made isolated flow chamber (tube with a length of 510 mm and inner diameter of 30 mm) with a gas flow rate of ~ 2 L/h (CH_4_ 21000 ppm, SO_2_ 50 ppm) and in a stationary mode [NO_2_7, 20 and 46 ppm, acetone (liquid evaporation) 7000 ppm, ethanol (liquid evaporation) 7000 ppm]. The choice of the operating mode is related to features of the chamber construction and gas generation source.

For this study, single-electrode sensors were chosen as simple and cheap bases for the general characterization of the composite characteristics. It should be noticed, that the construction of the sensor allows fast and easy maintenance and conduction of basic sensing measurements, however, is not suitable for commercial use because of higher operation temperatures and short operating time. A detailed description of the sensor construction and operating principles could be found elsewhere^19,37^.

The operating current was supplied from a B5-49 direct current power supply; the measurement conditions and sensor parameters were controlled by a B7–40/4 multimeter. The output signal (sensing response) of single-electrode sensors was determined as U = U_air_ − U_g_, where U_air_ and U_g_ are the voltage on the sensors at a constant heater current in the air and a gas mixture, respectively. The temperature of the sensitive elements of single-electrode sensors was measured using a laser pyrometer with an Impac IN140 micro-target designator (LumaSense Technologies) (Fig S4). The pyrometer enables the determination of the temperature of point sources of radiation > 200 °C. The response of the sensors was measured in the range of 80–160 mA (~ 220 to 500 °C).

## Results and discussion

The sol–gel approach used for the synthesis of the In_2_O_3_–G@Cu composites in this research was employed before for obtaining pure oxide and complex nanomaterials like WO_3_, WO_3_-In_2_O_3_, WO_3_–Co_3_O_4,_ and WO_3_–graphene–Cu composites^21,25,38^. In the latter case, the study showed, that the introduction of the graphene-based additive into sol with subsequent in situ oxide-based composite formation results in good distribution of the G@Cu on the surface of the tungsten oxide and influences its microstructure, near-to-surface chemical composition, surface, and bulk characteristics and composites gas sensing properties. Thus, for a better understanding of the nature of the In_2_O_3_–G@Cu properties, it is important to study the features of the microstructure of the composites, their phase composition, and the effect of the interaction between the oxide and the G@Cu.

### Materials characterization

**Figure 2:**
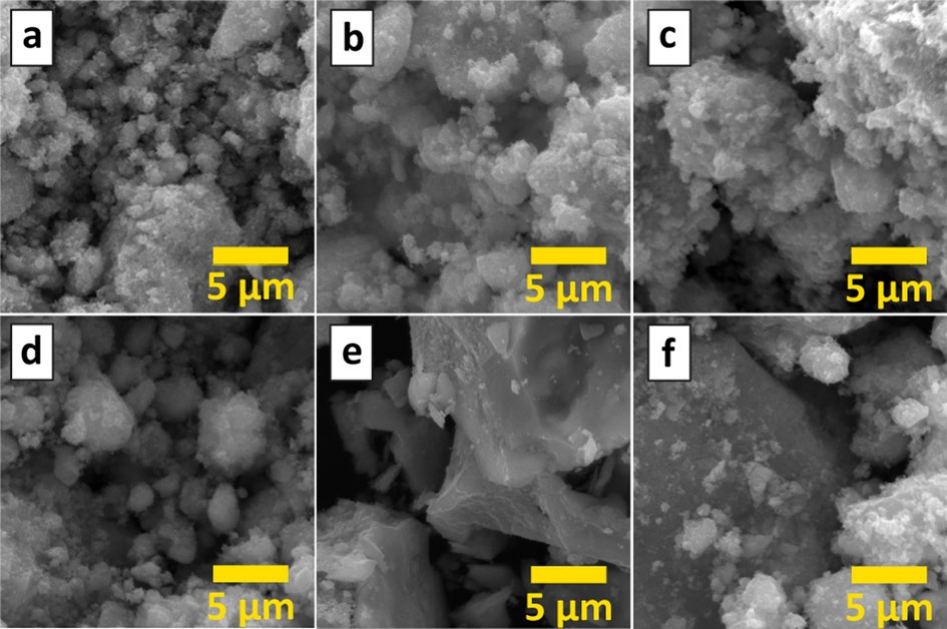
SEM images of the synthesized powders of the (**a**) IG0, (**b**) IG1, (**c**) IG2, (**d**) IG3, (**e**) IG4, and (**f**) IG6 samples.

The examples of the typical microstructure of IG powders and EDS elements mapping are shown in SEM images in Fig. 2 and Fig. S5.

From the images, it could be seen, that all powders consist of irregular shape agglomerates of rounded smaller size grains of 0.5–5 μm. However, grains of more complex shapes also could be found. Such a combination of grains and agglomerates creates a microstructure with a highly developed surface. For instance, the BET surface area of pure In_2_O_3_ is ~ 60 m^2^/g, and for modified samples specific surface area increases gradually to 71–83 m^2^/g depending on G@Cu content. It should also be noticed that the sizes of observed agglomerates increase gradually from the smallest for pure indium oxide IG0 (Fig. 2a) to larger, up to several tens of micrometers, in the samples with higher additive contents IG1–IG-6 (Fig. 2b–f), which could be related to the bonding effect of graphene films, adsorbed on surfaces of the oxide grains and compensating excessive surface energy.

The results of the EDS elements mapping analysis (Fig. S5) confirm the main elements in all compositions are indium and oxygen. These two elements are the only ones that were found in meaningful quantities in the IG0 sample. For the samples with 1–6% of the G@Cu additive, C and Cu atoms, evenly distributed in powders volume, were also detected. Such a good elements distribution indicates that the applied sol–gel synthesis approach allows the creation of homogeneous complex G@Cu-containing composites, even with an additive content of only 1 wt%. The EDS evaluated the content of the Cu phase in the samples increased from 0.8 wt% in the IG1 sample, to 1.3 wt%, 1.6 wt%, 1.8 wt%, and 2.1 wt% for the IG2, IG3, IG4, and IG6 samples, respectively. The content of the carbon atoms also gradually changes in the same samples row reaching 4.1 wt% in the IG6 sample.

**Figure 3:**
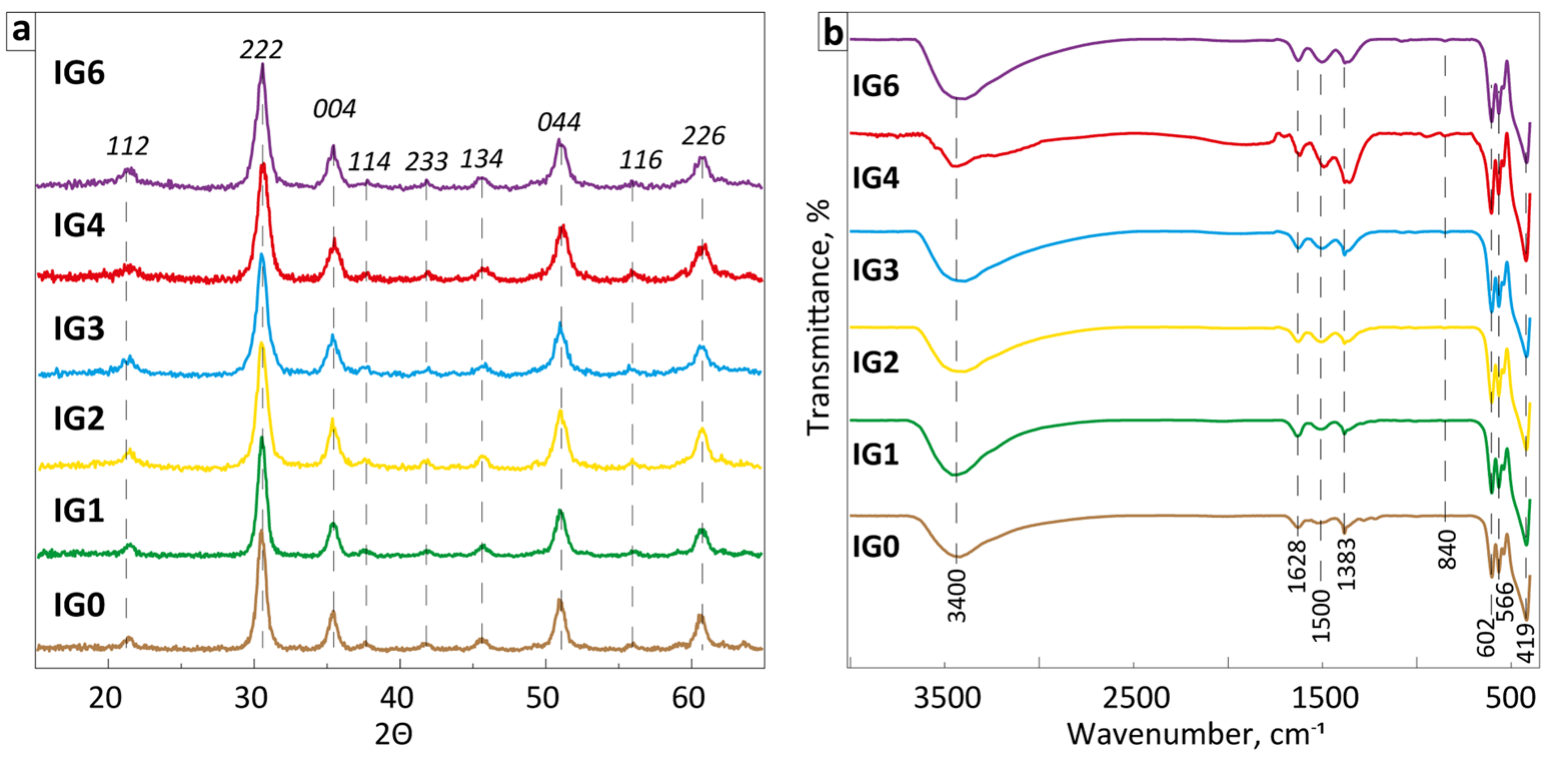
Typical normalized (**a**) XRD patterns and (**b**) FTIR spectra (wavenumber range 3700–400 cm^−1^) of the experimental samples.

To study the materials phase composition and features of their crystalline structures the XRD and FTIR spectra analyses were carried out. The results of the study are shown in Fig. 3a,b and presented in Table 1.

**Table 1:** Calculated phase compositions, cell parameters, and crystalline sizes of the samples.

Samples	Space group	Crystalline parameters		Crystalline size d, nm	Goodness of fit
		a, Å	Cell volume, Å^3^		
IG0	Ia-3 Cubic	10.1122	1034.035	10	0.79
IG1		10.1051	1031.855	11	1.05
IG2		10.1062	1032.195	8	1.01
IG3		10.1068	1032.387	7	1.10
IG4		10.0974	1029.501	8	0.80
IG6		10.0928	1028.086	8	1.44

The analysis of the XRD pattern of the IG samples (Fig. 3a) revealed that all obtained materials are crystalline In_2_O_3_ phase with cubic structure (Ia-3 space group). The main peaks in XRD patterns for all samples at around 21.40° (112), 30.60° (222), 35.45° (004), 37.77° (114), 41.81° (233), 45.62° (134), 50.90° (044), 55.86° (116), and 60.81° (226) are in a good agreement with the cubic In_2_O_3_ phase (JCPDS card no. 71-2194). Despite clear evidence of the presence of C and Cu in the compositions of the materials shown by the EDS analysis (Fig. S5) peaks of any other secondary crystalline phases, including G@Cu were not detected. This is likely due to the relatively low content of the G@Cu composite in the In_2_O_3_ matrix phase, as, for instance, even in the IG6 sample amount of separate Cu or G phases does not exceed 3 wt%, which is on the detection limit of the instrument.

The formation of the In_2_O_3_ was also confirmed by the FTIR spectra analysis in all cases (Fig. 3b). Four characteristic peaks of the cubic In_2_O_3_ phase at around 419, 566, 602, and 840 cm^−1^ were found in the spectra of all samples. According to the previously reported results^39,40^, the observed peaks at 419 and 566 cm^−1^ could be assigned to the In–O stretching band in the In_2_O_3_ cubic structure, as the peaks at 602 and 840 cm^−1^— to In–O bending vibrations in indium oxide, indicating the complete oxide crystalline phase formation. The peaks at around 1383, 1500, 1628, and 3400 cm^−1^ could be assigned to the nitrate group, an asymmetric stretching band of the carbonate group, bend deformation of water, and adsorbed OH^–^, respectively^40–42^.

Overall, based on the analysis of the XRD patterns and FTIR spectra we can conclude, that in all cases the In_2_O_3_ phase is characterized by a good crystallinity with no or very small amount of amorphous phase. It should be noticed that the crystalline cell parameter **a** is the largest for pure indium oxide, as an introduction of even 1 wt% of the G@Cu additive leads to a noticeable decrease of the **a** with a clear tendency for further crystalline cells shrinkage at higher additive content (Table 1).

**Figure 4:**
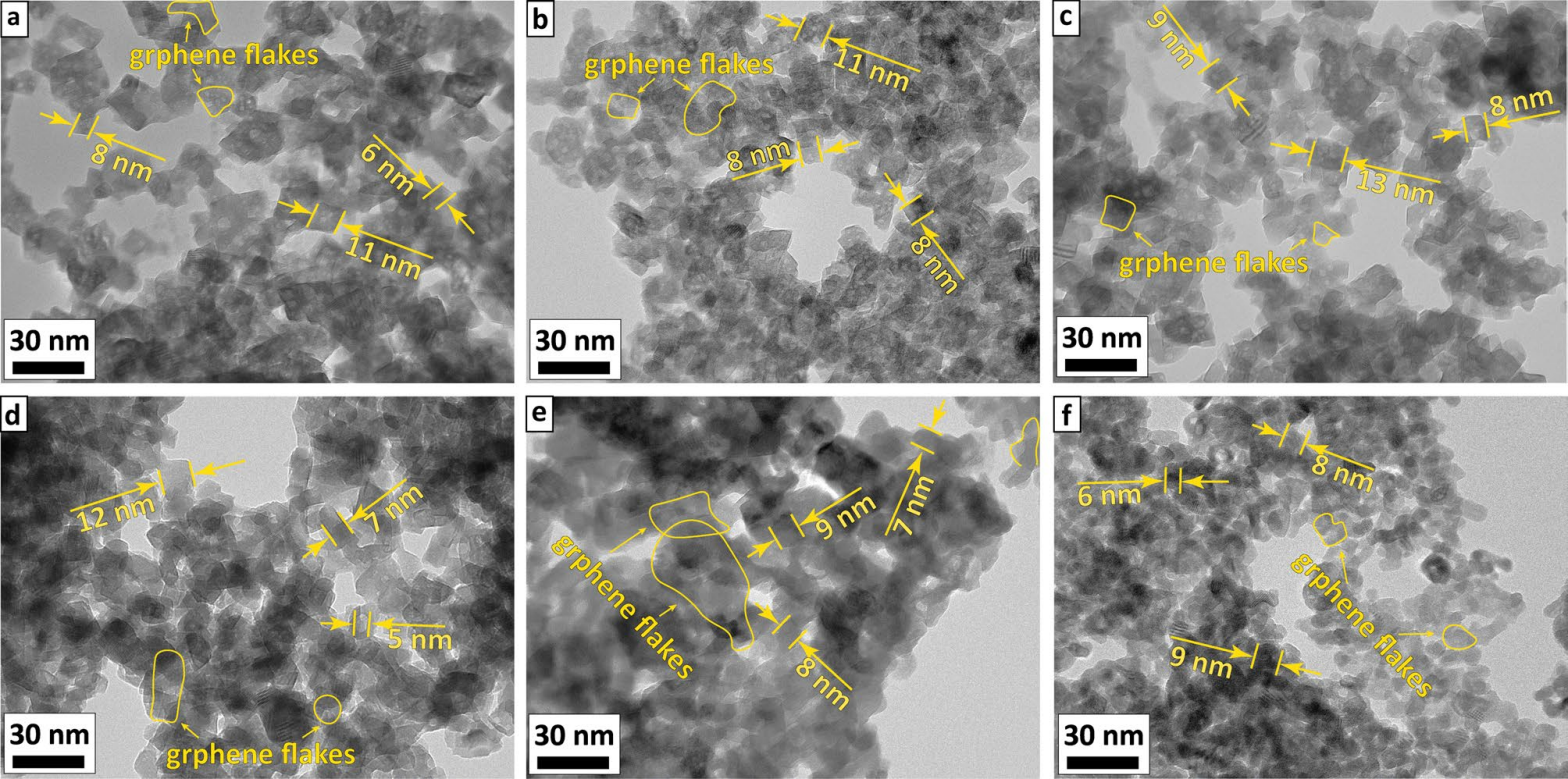
TEM images of the (**a**) IG0, (**b**) IG1, (**c**) IG2, (**d**) IG3, (**e**) IG4, and (**f**) IG6 samples.

A similar trend could be seen for the calculated values of the powders crystalline sizes – a decrease from 10 nm for the IG0 to 7–8 nm for the IG2-IG6 samples. The data corroborates the analysis of the TEM images of the powders of all compositions (Fig. 4a–f). From the images one could see, that the powders consist of small (5–13 nm) cubic crystals of In_2_O_3_ with clear edges, covered with graphene flakes. The In_2_O_3_ crystallites tend to decrease in size upon increasing G@Cu content. This trend, however, is accompanied by an increase in the degree of particle agglomeration, observed in SEM images (Fig. 2), as the result of increasing uncompensated surface free energy. We suppose the phenomena are related to the effect of the G@Cu on processes of indium oxide crystallization and crystal growth during sol maturation and xerogel calcination. Previously we showed^21^, that the presence of the G@Cu flakes in the sol precursor leads the highly-developed surface of G to become centers for oxide crystallites formation with their subsequent growth. In this case, the two structures (e.g. oxide and G@Cu) are closely interconnected and the G@Cu plays the role of a surface terminator, which limits the ability of the oxide crystals to grow, decreasing crystallites sizes. We suppose, that the addition of G@Cu flakes during the maturation stage of In-based gel leads to the formation of an interconnected system, where G@Cu flakes act as centers for the initial nucleation of In_2_O_3_. This process is likely driven by the energy gain resulting from the compensation of the excessive surface energy of the G@Cu flakes by newly formed In_2_O_3_ crystallites. Additionally, the energy factor may limit the growth of the oxide crystallites, making it less energy beneficial for them to grow beyond the size of the flakes compared to the nucleation of new crystallites on the free surface of the G@Cu flakes. Furthermore, we suppose the close interconnection of the oxide and graphene phases leads to mechanical compression of the In_2_O_3_ crystal cell by G@Cu flakes, resulting in crystalline cell shrinkage.

**Figure 5:**
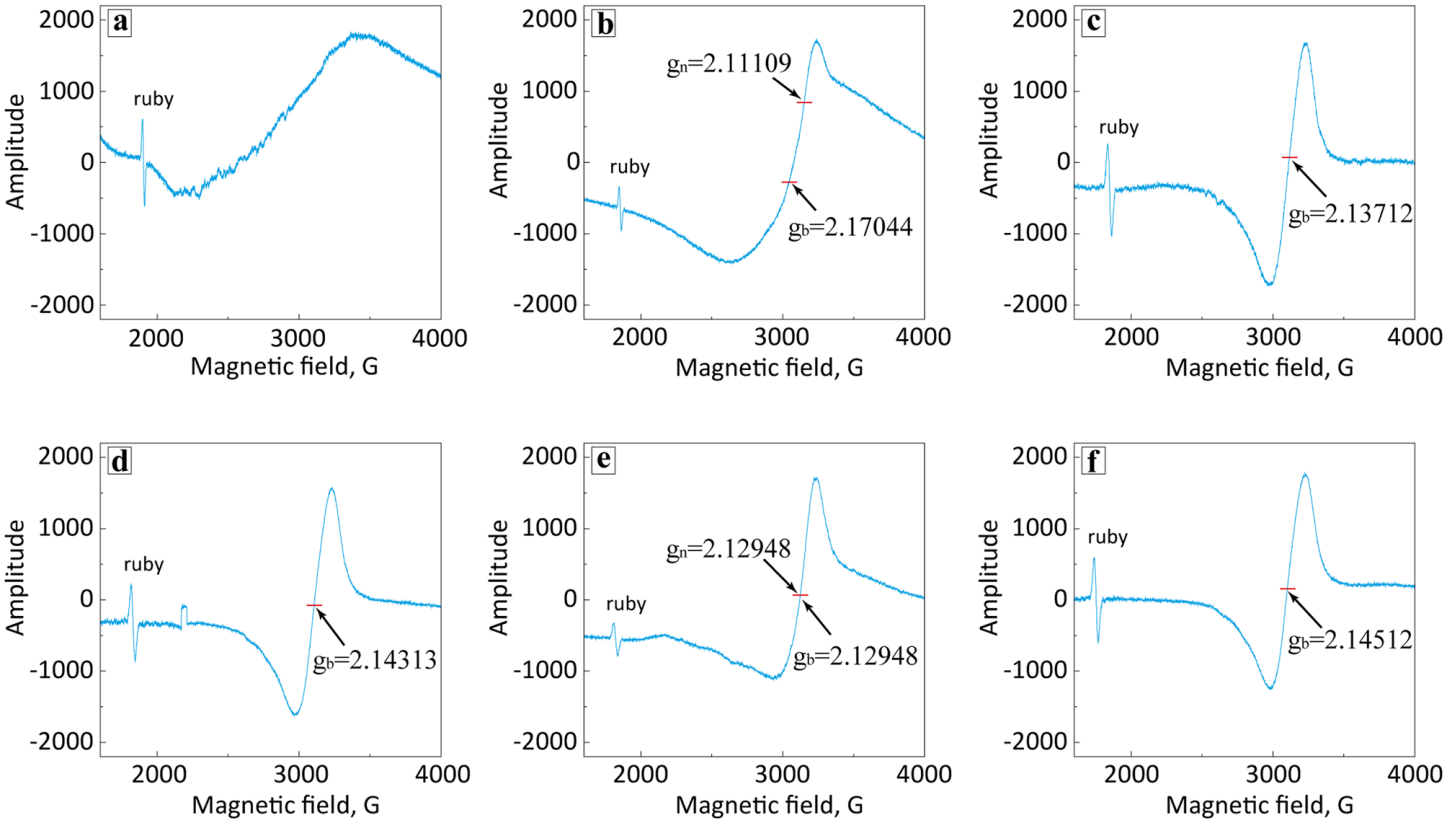
Room-temperature EPR spectra of the (**a**) IG0, (**b**) IG1, (**c**) IG2, (**d**) IG3, (**e**) IG4 and (**f**) IG6 samples.

Further understanding of the influence of G@Cu modification on the structure of In_2_O_3_ can be revealed by analyzing the EPR spectra of the samples (Fig. 5; Table S1).

From the data, it can be seen, that the spectrum of pure indium oxide shows no EPR signal, indicating it is EPR inert at room temperature (Fig. 5a). This confirms that pure indium oxide is a highly stable well crystalline phase with low defectiveness in its structure. On the other hand, the spectra of all samples modified with the G@Cu nanocomposite exhibit one (IG2, IG3, and IG6 (Fig. 5c,d,f)) or two (IG1 and IG4 (Fig. 5b,e) types of EPR signals corresponding to broad and narrow peaks. The signal amplitude, which is proportional to the defects concentration, of broad peaks is the highest for the IG3 sample, while the most intensive narrow peak was detected for the IG4 powder, containing 4 wt% additives. In the EPR spectrum of the IG6 sample, there was detected an additional very broad EPR signal, which is a sign of paramagnetic centers with a strong exchange interaction of uncompensated electron spins. Such an interaction is characteristic of magnetically ordered systems.

Based on the presented results we can assume the formation of weak bonds between In_2_O_3_ crystals and G@Cu nanocomposites, which, in its turn, cause the formation of oxygen vacancies and other structural defects. However, an increase in the thickness of the G@Cu adsorbed layer on the oxide surface leads to partial compensation of the structural inhomogeneity with a saturation effect at ~ 6 wt% when the adsorption of an additional amount of the graphene–metal composite accumulates on the In_2_O_3_ surface without a significant effect on the structural defectiveness. The appearance of the broad peak in the EPR spectra of modified In_2_O_3_ can also be associated with a change in the chemical state of indium oxide due to the modification of the oxide crystals with G@Cu. It should be noted, that signals in all modified samples are characterized by g-factor values close to the ones of free electron (2.000). Such g-factor values along with the shape of the peaks indicate the possible presence of hole centers and their associates, as well as F-centers in the modified In_2_O_3_ structure^25^. Therefore, structural distortion causes the presence of paramagnetic centers that are similar in nature but have different environments or different charge states.

Special attention should be drawn to the IG4 sample, containing 4 wt% of G@Cu nanocomposite additives. Based on the results of the EPR spectra analysis we can conclude, that the combination of indium oxide with carbon and copper atoms, in this case, leads to the formation of structures, which is conductive in the microwave range. This is confirmed by a significant decrease in the resonant frequency and amplitude of the reference sample that controls the quality factor of the resonator. A decrease in the values of these parameters is indicative of non-resonant losses in the resonator due to the conductive structures formed during the modification of the In_2_O_3_. Such structure is usually very sensitive to even small surface modifications or interactions with the inner atmosphere and therefore is a promising candidate for application as a gas sensing material. The specific reason for the formation of such a structure and why it was not observed in other materials are not clear and require additional study.

Overall the EPR data indicate an increase in the concentration of paramagnetic defects in all modified samples compared to pure In_2_O_3_. The formation of a highly defective structure is a prerequisite for the manifestation of a high gas sensitivity of materials since structural defects are centers of the adsorption of gases of various types.

### Sensing properties

**Figure 6:**
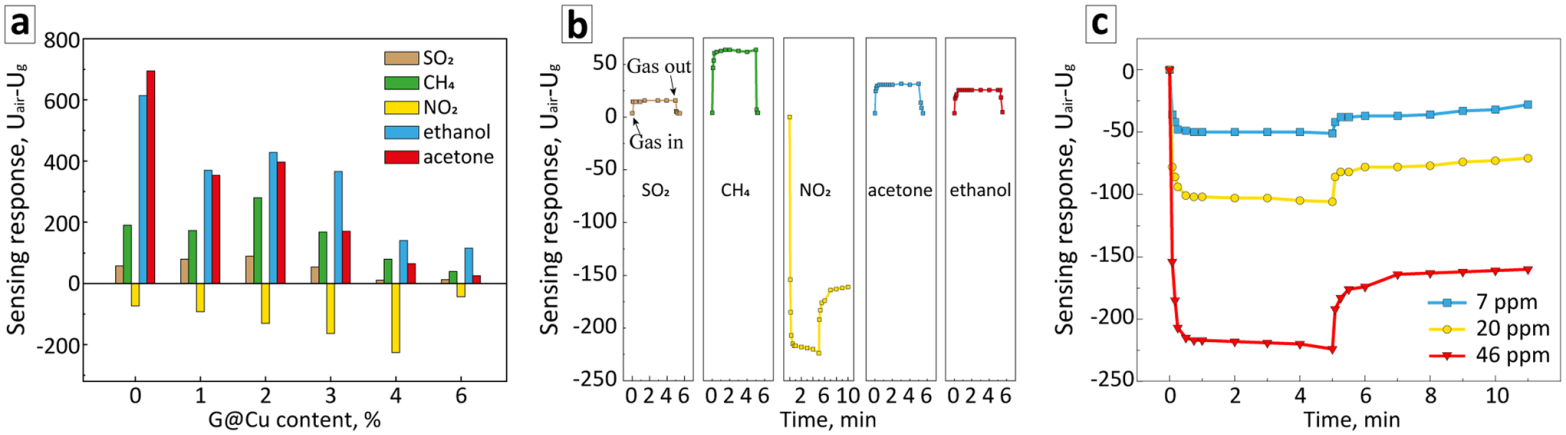
(**a**) Gas sensing response of single-electrode sensors with IG composites as sensing elements to different test gases at optimal temperatures. (**b**) Sensing response of IG4 sensor to different test gases at the heating current of 131 mA (~ 420 °C). (**c**) Sensing response of IG4 sensor to gas mixtures with various concentrations of NO_2_ at the heating current of 131 mA (~ 420 °C).

Gas sensing tests were performed on single-electrode semiconductor gas sensors using five types of gases: oxidizing NO_2_, reducing SO_2_, and VOC methane, acetone, and ethanol, which also have reducing properties. The results of measurements of the sensors' characteristics are presented in Fig. 6, Figs. S6, and S7, and Table S2.

The study of the sensitivity of the sensing elements with the different materials compositions in the atmosphere of different gases in conditions of increasing heating current showed that the sensitivity of sensors with all experimental compositions is temperature-induced. In all cases, there are ranges of heating current with a clear peak in sensing response values of sensors (Figs. S6, S7). In general, the working heating current values for sensors with sensing elements based on IG composites are within the range of 91–161 mA (~ 280 to 510 °C) with a tendency for an increase in the working heating current of sensors with an increase of G@Cu content in IG compositions. However, the heating current values inducing a noticeable sensing response are materials and gas-specific (Figs. S6, S7). For instance, acetone and ethanol show the highest sensing response of 695 mV and 614 mV, respectively, in the case of an IG0 sensor (Fig. 6, Table S2), as an increase of the G@Cu content in the IG composites leads to a significant decrease in sensing response to these two gases. In the case of other reducing gases, however, the initial increase in the G@Cu content from 0 to 2 wt% leads to an increase in the sensing response of sensors for ~ 35 and ~ 32% from 58 to 90 mV for SO_2_ and from 191 to 280 mV for CH_4_, respectively. Further G@Cu content increase in In_2_O_3_-based composites leads to a systematic decrease in sensing response with the lowest values of 13 mV and 40 mV for SO_2_ and CH_4_, respectively, in the case of the IG6 sensor.

Remarkably different behavior of sensors was observed in the case of NO_2_. The sensing response systematically increases upon an increase in G@Cu concentration from 73 mV for a sensor with IG0 composite, to −92, 130, 163, and 225 mV for sensors with IG1, IG2, IG3, and IG4 composites, respectively, while the sensor with the IG6 composite shows the lowest sensing response for NO_2_ of 43 mV. It should be noticed, that the trend in sensitivity change, as well as the peak value of the sensing response to the NO_2_, corroborates with the EPR data (Fig. 5; Table S1) that shows the systematic increase in the concentration of structural defects in raw of samples from IG0 to IG4. The latest predictably shows the highest sensing response as it is the most susceptible to a change in charge and chemical state of its surface as the result of gas molecules adsorption. Furthermore, in the case of the IG4 sensor, the sensing response to NO_2_ is the highest in comparison to the absolute values of the response to all other tested gases. We suppose the selectivity to the NO_2_ could be related to Cu and graphene acting as reducing agents, altering the redox potential of the system in a manner that increases its attraction for oxidizing gases. In the case of IG6, however, the formation of the magnetically ordered system founded by the EPR data analysis may indicate the decrease of the number of near-to-surface structural defects, related to the saturation with the G@Cu nanocomposite and causing a sharp decrease in gas sensitivity.

We chose the sensor with IG4 composite as a sensing material for further study of its sensing characteristics.

Figure 6b shows the time-resolved profiles of the sensing response of the IG4 sensor to the test gases. It could be seen, that in all cases the sensor is characterized by a very fast response time of about 5–15 s with a stable signal level after reaching saturation. However, the recovery behavior of the sensor differs between reducing and oxidizing gases. Both SO_2_ and methane show a very fast recovery in less than 10 s. The recovery time for acetone and ethanol of 30 s is slightly higher but still relatively fast. On the other hand, in the case of nitrogen oxide, full recovery of the initial sensor’s signal was not observed until the heating current was reduced or switched off. Similar behavior was also observed in the case of measurements of sensing response to NO_2_-based gas mixtures with nitrogen oxide concentrations of 7 and 20 ppm (Fig. 6c). In cases of gas mixtures of all three NO_2_ concentrations, the sensor shows a similar optimal operating heating current (temperature) range with the maximal sensing response at ~ 131 mA (Fig. S8). Regardless of the NO_2_ concentration applied, the sensor reaches saturation in less than 10 s and shows a stable saturation signal, while the recovery value is only about 30%. The phenomenon was reported for graphene-based sensors before and is possibly related to the chemical reaction of nitrogen oxide on the graphene surface^43^. It should also be noted, that the IG4 sensor shows a very good linear dependence of the sensing signal from the concentration of nitrogen oxide with a coefficient of determination of 0.99955 (Fig. S9).

The In_2_O_3_–G@Cu nanocomposites are complex materials formed as a combination of In_2_O_3_ n-type semiconductor, p-type highly defective SCS-graphene films/flakes similar to rGO and surface oxidized metallic copper nanograins, distributed into the graphene film^36,44^. The composite nanostructure creates a complex system of closely interconnected elements of different natures and a highly defective nano- and microstructure. Such a system is gradually more complex than a single oxide or even a simple combination of two semiconductors of the same or different types. One of the commonly accepted explanations for the increase in the sensing response and decrease in the operating temperature of the oxide-graphene-based sensors in comparison with the pure oxide is related to the formation of interconnected structure on the oxide-graphene grains boundaries between the n- and p-type indium oxide grains and graphene flakes adsorbed on their surfaces, respectively. In such a structure, electrons tend to move from the graphene toward the oxide surface, which leads to the formation of negatively and positively charged regions^45^. Moreover, the formation of closely interconnected nanostructures between In_2_O_3_ and G@Cu composites could lead to the formation of weak In–O–C bonds at the interphase boundaries, analogous to bonds, found for WO_3_–graphene composites^46^. The chemical bonding could increase the efficiency of charge carrier transfer between semiconductors of different types and facilitate the composite sensing response^22,47^.

Another important factor, that affects the sensing mechanism is the state of the sensing surface and the type of adsorbed gas molecules. The surface of oxides usually contains a variety of chemisorbed negatively charged oxygen adions, like O^−^, O_2_^−^, and O^2−^. The oxygen adions form as a result of the adsorption of oxygen molecules from the air and surface charge transfer. This process promotes the formation of an electron-depleted layer with a potential barrier on the oxide surface. The stability and reactivity of different forms of oxygen adions depend on the surface energy and specific properties of the semiconductor^48^. For instance, the highly-defected graphene flakes, adsorbed on the oxygen semiconductor surface, could facilitate the formation of the adions due to the catalytic effect. In addition, graphene sheets with a large available surface area create a hierarchical nanostructure and facilitate gas molecule diffusion, increasing the number of surface contacts and enhancing the gas chemisorption reaction. Direct interaction of molecules of the test gases with the negatively charged adions affects the surface layer electron density of the materials resulting in a change in their surface conductivity. For instance, strongly oxidizing molecules of NO_2_ could interact with the chemisorbed oxygen or capture free electrons from the conduction band of the semiconductor surface directly. In both cases, the NO_2_ molecule transforms into a negatively charged NO_2_^−^. This causes the electron depletion layer thickness increase, accumulation of holes, and leads to a decrease in the conductivity, i.e. increase in gas sensing response, of the indium oxide surface^49,50^. Furthermore, the accumulation of electrons transferred from the surface of n-type semiconductor indium oxide in the p-type semiconductor G-phase facilitates charge exchange between gas molecules and the graphene surface. The nitrogen oxide molecules play the role of electron acceptors and hole donors, thereby restoring the hole concentration and p-type conductivity of graphene. The charge transfer process becomes possible in this case, because of the alignment of the energy bands between the oxide and graphene. Previously it has been shown^46^ that interaction with oxidizing gas molecules could lead to upward band bending and shifting the Fermi level of the oxide semiconductor toward the valence band. This, in combination with electron capturing by NO_2_ molecules, facilitates the electron–hole exchange on the indium oxide-graphene interface leading to a higher degree of electron depletion of the conduction band of the indium oxide and a general decrease in sensing material conductivity. Furthermore, an initial gas molecules' electron functionalization by interaction with oxygen adions and sensing material surface could also play the important role in the charge transfer.

On the other hand, the interaction of reduction-type gases, like SO_2_, CH_4_, acetone, and ethanol with the chemisorbed oxygen adions leads to an appearance of free electrons, that transfer into the conduction band of the semiconductor^48,51^. In this case, the processes that occur on the surface of the sensor could be described as follows (Eqs. 1,2,3,4):

1 $${\text{SO}}_{{{2}({\text{gas}})}} + {\text{O}}^{ - }_{{({\text{surf}})}} \to {\text{SO}}_{{{3}({\text{gas}})}} + {\text{e}}^{ - }$$

2 $${\text{CH}}_{{{4}({\text{gas}})}} + {\text{4O}}^{ - }_{{({\text{surf}})}} \to {\text{CO}}_{{{2}({\text{gas}})}} + {\text{2H}}_{{2}} {\text{O}} + {\text{4e}}^{ - }$$

3 $${\text{C}}_{{2}} {\text{H}}_{{5}} {\text{OH}}_{{({\text{gas}})}} + {\text{6O}}^{ - }_{{({\text{surf}})}} \to {\text{2CO}}_{{{2}({\text{gas}})}} + {\text{3H}}_{{2}} {\text{O}} + {\text{6e}}^{ - }$$

4 $${\text{CH}}_{{3}} {\text{COCH}}_{{{3}({\text{gas}})}} + {\text{8O}}^{ - }_{{({\text{surf}})}} \to {\text{3CO}}_{{{2}({\text{gas}})}} + {\text{3H}}_{{2}} {\text{O}} + {\text{8e}}^{ - }$$

As a result, electrons that were trapped in the oxygen adions return to the conduction band of indium oxide, and its conductivity increases, i.e. increase gas sensing response. We suppose, in this case, the p-type semiconductive highly-defected graphene species play a major role in the facilitation of electron transfer from adsorbed reducing type gases, oxygen adions, and surface functional groups to the inner graphene structure, compensating for excessive holes concentration. This, in its turn, enhances the transition of electrons from the adsorbed gas molecules to the conduction band of indium oxide and enhances the sensitivity of the material surface to the adsorbed gas molecules.

Another important factor, that possibly affects the sensing behavior of the IG composites is the presence of copper/copper oxide nanograins. Metallic and metal oxide nanoparticles in general and copper, in particular, are considered as advanced heterophase catalysts^36,52,53^. Thus, we suppose one of the main roles of the copper-based nanograins is the facilitation of selective catalysis of oxygen molecules for the formation of adions and, also reactive transformation of gas molecules on the sensing material surface according to processes, described early. Furthermore, it is known^54,55^, that semiconductors with a high potential barrier tend to form a wide depletion zone as a result of contact with the metallic nanoparticles with large electrons work function such as Ag (4.7 eV), Pd (5.0 eV), Pt (5.3 eV), and Cu (4.68 eV) — the spillover effect^56^. The decrease in a potential barrier height at the metal–semiconductor interfaces facilitates the electron transfer from metallic nanoparticles to graphene and indium oxide and leads to a general increase in surface conductivity. This could be related to the strong distortion of the carbon π-orbitals in graphene and the formation of graphene edge defects due to its interaction with copper grains and carbon π-orbitals in graphene^57^. The efficiency of the mechanism depends on the concentration and degree of distribution and oxidation of the copper nanograins on the graphene surface, and temperature. The effect could be enhanced by the formation of the closely interconnected nanocomposite structure, obtained as a result of sol–gel synthesis, where G@Cu was added in the stage of gel formation. However, the role of copper particles remains to be further clarified.

In addition, the effect of G@Cu composite introduction can be enhanced by a large specific surface area of the material^21,33^.

## Summary

In this research, the In_2_O_3_-based materials, modified with 0–6 wt% of the SCS obtained graphene–Cu nanocomposite, were prepared by sol–gel method, carefully characterized, and studied as sensing materials in single-electrode semiconductor gas sensors. It was found that (1) the introduction of the G@Cu nanocomposite into In-containing gel precursor leads to the formation of a closely interconnected and highly defective In_2_O_3_–G@Cu complex structure and terminates indium oxides crystal growth; (2) the defectiveness of the crystal structure increases upon an increase in the G@Cu content up to 4 wt% with the subsequent saturation as sizes of the In_2_O_3_ crystallites gradually decreases from 10 nm for pure oxide to 7–8 nm for modified one; (3) the nanocomposites show high sensing response to both oxidizing (NO_2_) and reducing (acetone, ethanol, methane) gases at an optimal working heating current of 91–161 mA (280–510 °C) with the saturation and recovery time of about 10–30 s with an exception of NO_2_ with no full recovery observed until heating current reduced or switched off; (4) the sensor with nanocomposite with 4 wt% of G@Cu additive to In_2_O_3_ showed the highest sensitivity to NO_2_ (46 ppm) in comparison with other tested gases with an absolute value of sensing response of (−) 225 mV and linear dependence of sensing response to NO_2_ concentration; (5) the major factors influence the sensing properties of the In_2_O_3_–G@Cu nanocomposites are the formation of available for efficient gas adsorption highly defected and closely interconnected semiconductor structure of n-type indium oxide and p-type defected SCS graphene with a high free electron concentration and relatively low potential energy barriers for electron transitions, and the additional catalytic effect of the graphene and Cu/CuO phases.

Overall, the obtained nanocomposites have great research interest and potential for practical application in highly sensitive gas sensors of gases of different chemical natures. The proposed synthesis approach could also be used for obtaining a broad variety of nanomaterials and compositions, with controllable phases and microstructures.

## Supplementary Information

Supplementary Information.

## Data Availability

The datasets used and/or analyzed during the current study are available from the corresponding author upon reasonable request.
