# Supplementary material for: High-performance selective NO2 gas sensor based on In2O3–graphene–Cu nanocomposites
Source: Sci Rep. 2023 May 15;13:7834. doi: 10.1038/s41598-023-34697-5 (PMC10185554; doi:10.1038/s41598-023-34697-5)
Supplement: Supplementary file 1 — Supplementary Information. [file 41598_2023_34697_MOESM1_ESM.pdf]

Supporting Information for

High-performance selective NO_2 gas sensor based on In_2O_3-graphene-Cu nanocomposites

Alexander Khort[a,*], Yulyan Haiduk[b,*], Igor Taratyn[c], Dmitry Moskovskikh[d], Kirill Podbolotov[e], Alexandra Usenka[b], Natalia Lapchuk[b], Vladimir Pankov[b]

[a]KTH Royal Institute of Technology, Drottning Kristinas vag 51, SE-100 44 Stockholm, Sweden
[b]Belarusian State University, Niezaleznasti av. 4, 220030, Minsk, Belarus
[c]Belarusian National Technical University, Prospekt Nezavisimosti, 65, 220013, Minsk, Belarus
[d]Center of Functional Nano-Ceramics, National University of Science and Technology MISIS, Lenin av. 4, 119049 Moscow, Russia
[e]Institute of Physics and Technology, Belarussian Academy of Sciences, Kuprevicha 10, 220141, Minsk, Belarus

*Corresponding authors: khort@kth.se (A. Khort), haidukys@bsu.by (Y. Haiduk).

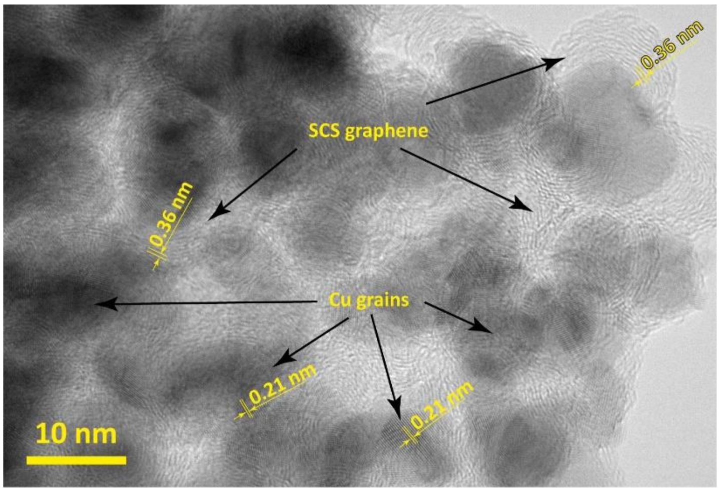

Figure S1. HTEM image of the G@Cu composite

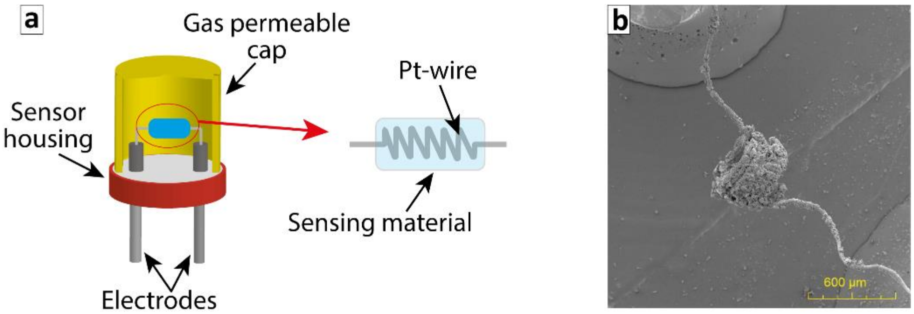

Figure S2. (a) Simplified scheme and (b) SEM image of single-electrode gas sensor

The principle of operation of single-electrode ceramic sensors is described in detail in (1, 2, 3). Such sensors are easier and cheaper to manufacture than planar sensors and could be used for research purposes. However, there are several commercials available on the market as well. There are several significant differences in operation principles between single-electrode sensors and the two-electrode design. In this case, the total resistance of the sensor is equal to the sum of the resistances of the platinum spiral and the ceramic layer, so the operating temperatures of single-electrode and two-electrode sensors do not coincide.

The maximum response is achieved at a certain ratio of resistances of ceramic material and electrode. To achieve this, it is necessary to reduce the resistance of the ceramic and the thickness of the platinum wire. However, the first condition does not guarantee a maximum response, and the second one reduces the reliability of the sensor.

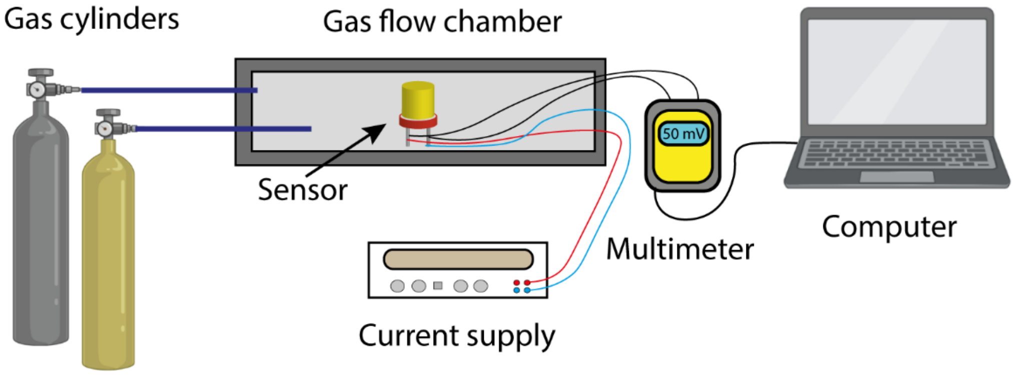

Figure S3. Simplified diagram of measurement system

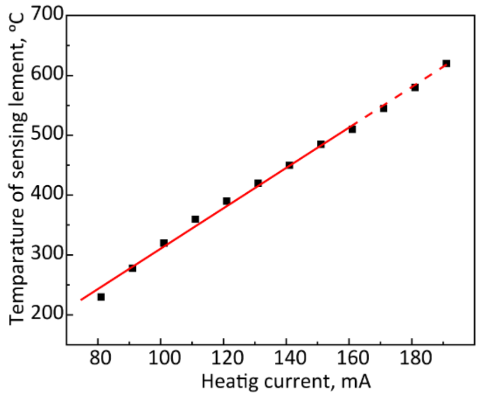

Figure S4. Heating current *vs* temperature calibration plot for single-electrode gas sensors

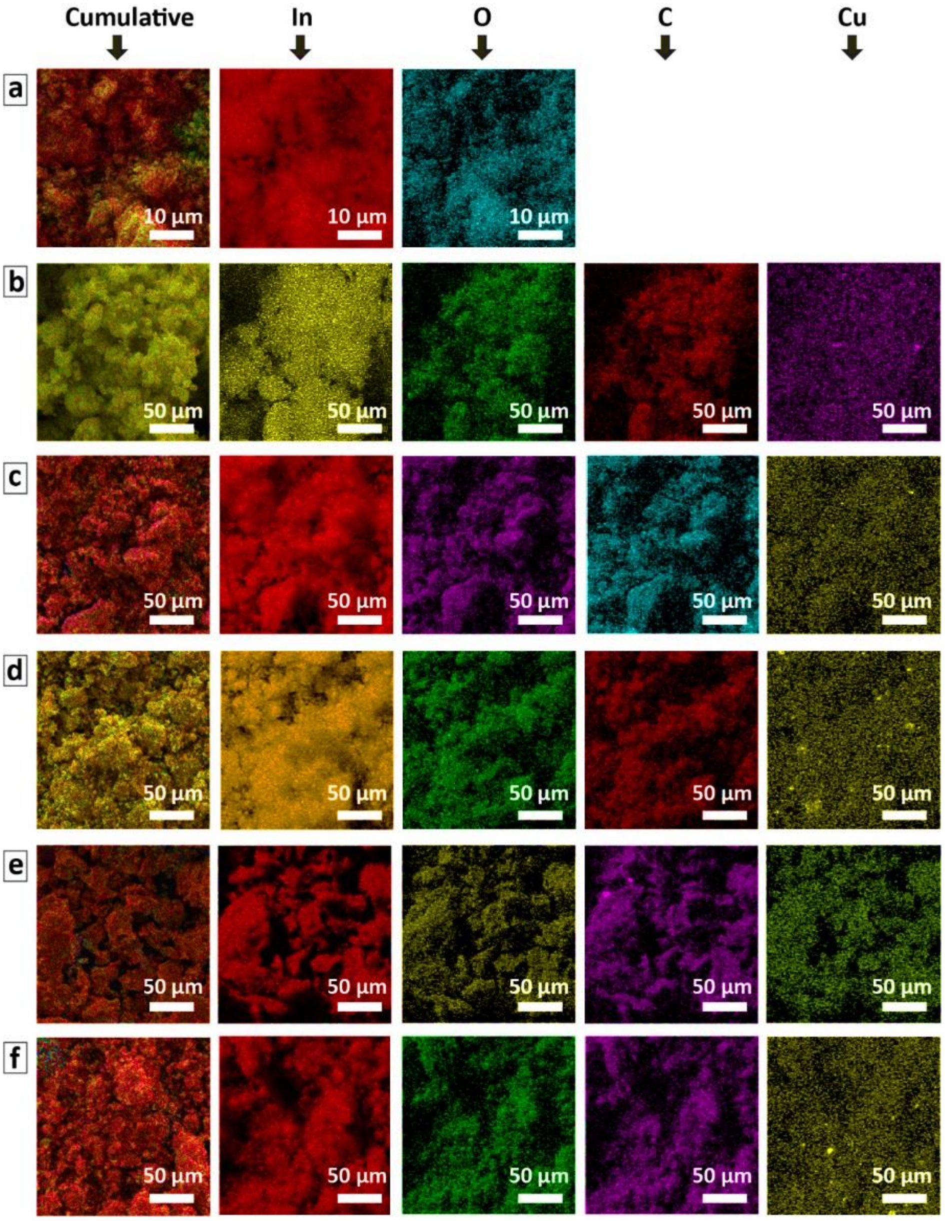

Figure S5. EDS elements mapping of the synthesized powders of the (a) IG0, (b) IG1, (c) IG2, (d) IG3, (e) IG4, and (f) IG6 samples

Table S1. Calculated parameters of the EPR spectra of In_2O_3-G@Cu composites.

Samples	g-factor	Resonance frequency, GHz	Line width, G	Amplitude, a.u.
IG0	No EPR peaks			
IG1	2.17044	9.3180445	580.53	2566.34 (b)
	2.11109		142.88	1459.62 (n)
IG2	2.13712	9.3187541	251.01	1690.52 (b)
IG3	2.14313	9.3152842	250.02	3193.31 (b)
IG4	2.12948	9.3092798	350.34	2192.08 (b)
	2.12948		201.34	3390.62 (n)
IG6	2.14512	9.3163178	242.37	3018.46 (b)

b – broad peak; n – narrow peak.

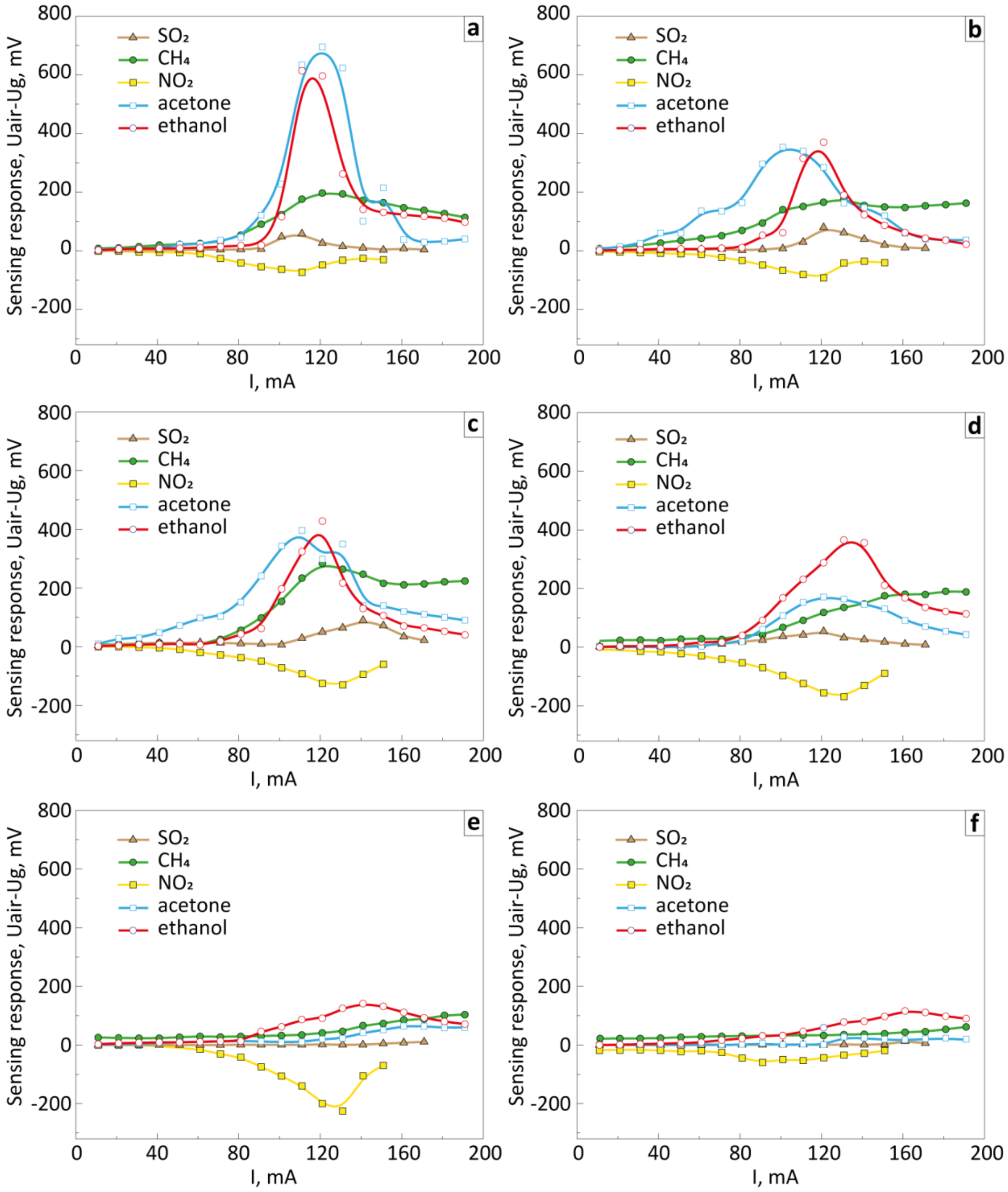

Figure S6. Heating current *vs* Sensing response to different gases of the single-electrode gas sensors with sensing elements based on (a) IG0, (b) IG1 (c) IG2, (d) IG3, (e) IG4, and (f) IG6 materials compositions

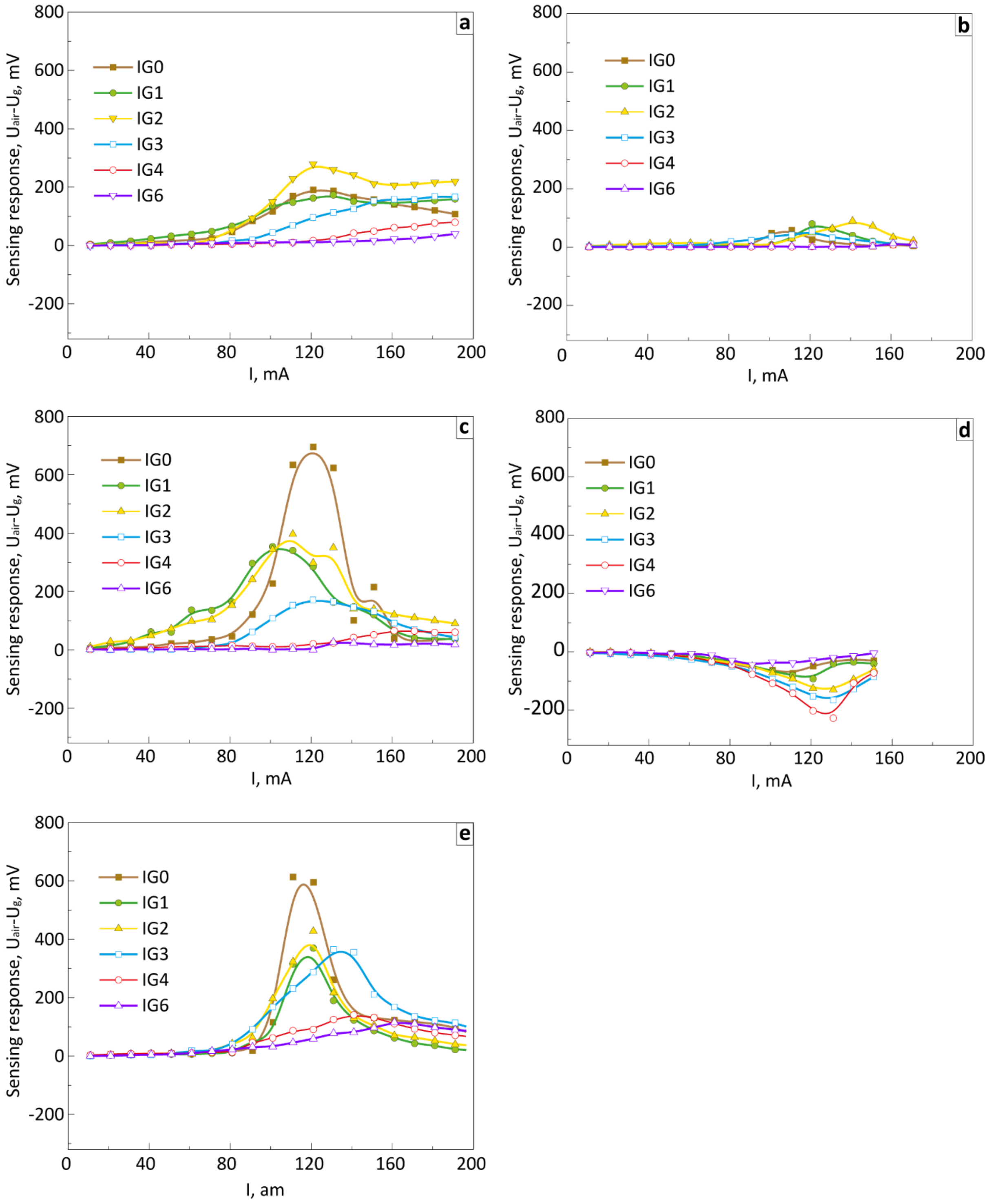

Figure S7. Heating current *vs* Sensing response of the single-electrode gas sensors with different compositions of the sensing elements in contact with (a) CH_4, (b) SO2, (c) acetone, (d) NO_2, and (e) ethanol

Table S2. Maximal sensory response of the sensors with different materials compositions to experimental gases, heating current on sensors, and corresponding temperature

Gases		Samples					
		IG0	IG1	IG2	IG3	IG4	IG6
SO_2	Max. sensory response, U_{air}-U_g, mV	58	80	90	54	11	13
	Heating current I, mA	111	121	141	121	171	161
	Temperature, °C	360	390	450	390	540	510
CH_4	Max. sensory response, U_{air}-U_g, mV	191	174	280	169	80	40
	Heating current I, mA	121	131	121	181	191	191
	Temperature, °C	390	420	390	580	620	620
NO_2	Max. sensory response, U_{air}-U_g, mV	-73	-92	-130	-163	-225	-43
	Heating current I, mA	111	121	131	131	131	91
	Temperature, °C	360	390	420	420	420	280
Ethanol	Max. sensory response, U_{air}-U_g, mV	614	370	428	365	141	116
	Heating current I, mA	111	121	121	131	141	161
	Temperature, °C	360	390	390	420	450	510
Acetone	Max. sensory response, U_{air}-U_g, mV	695	353	396	171	65	26
	Heating current I, mA	121	101	111	121	161	131
	Temperature, °C	390	320	360	390	510	420

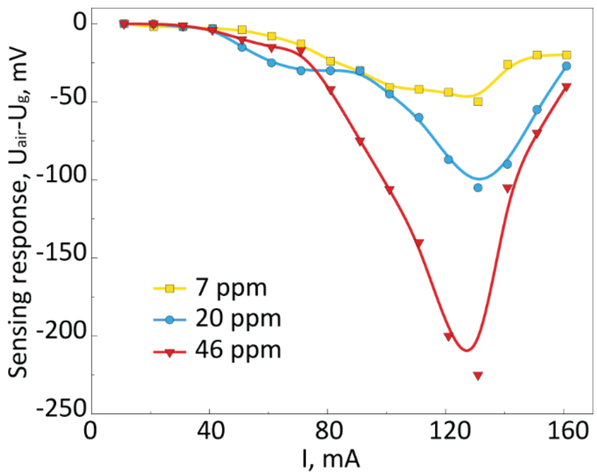

Figure S8. Heating current *vs* Sensing response of the single-electrode gas in contact with NO_2 of different concentrations

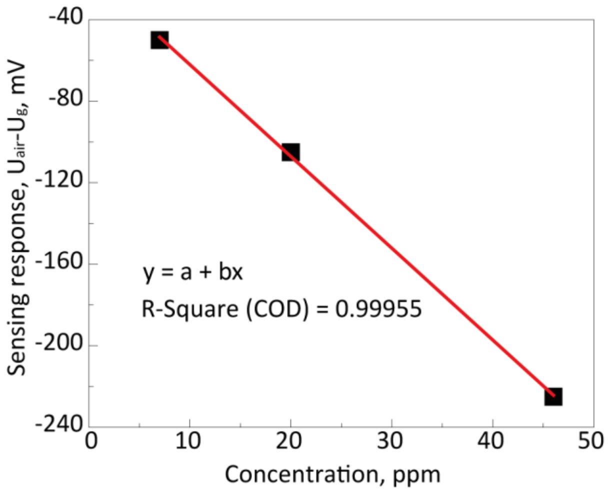

Figure S9. Concentration vs sensing response to NO_2 of the IG4 sensor

1. Korotcenkov G, Boris I, Brinzari V, Golovanov V, Lychkovsky Y, Karkotsky G, et al. Gas-sensing characteristics of one-electrode gas sensors based on doped In2O3 ceramics. Sensors and Actuators B: Chemical. 2004;103(1-2):13-22.
2. Korotcenkov G. Practical aspects in design of one-electrode semiconductor gas sensors: Status report. Sensors and Actuators B: Chemical. 2007;121(2):664-78.
3. Korotcenkov G, Boris I, Brinzari V, Han SH, Cho BK, Lychkovsky YN. In2O3:Ga and In2O3:P-based one-electrode gas sensors: Comparative study. Ceramics International. 2015;41(6):7478-88.
